# Long-term quality of life and functional outcomes in extracorporeal membrane oxygenation survivors

**DOI:** 10.1016/j.jhlto.2026.100590

**Published:** 2026-05-08

**Authors:** Salman Pervaiz Butt, Nabeel Razzaq, Salman Abdulaziz, Vivek Kakar, Daniel Brodie, Alain Combes, Hessa Al Shehhi, Alisa Higgins, Carol Hodgson, Umer Darr, Gopal Bhatnagar

**Affiliations:** a Manager, Perfusion Services & ECMO Specialist, Heart Vascular and Thoracic Institute, Cleveland Clinic, Abu Dhabi, UAE; b Perfusionist, Heart Vascular and Thoracic Institute, Cleveland Clinic, Abu Dhabi, UAE; c Critical Care Services Administration, King Fahad Medical City, Riyadh, KSA; d Consultant of Cardiovascular Critical Care, Chair of ECMO Program, Cleveland Clinic Abu Dhabi, UAE; e Professor, Professor of Medicine, Division of Pulmonary & Critical Care Medicine, The Johns Hopkins University School of Medicine, Baltimore, Maryland; f Professor of Intensive Care Medicine, Head of Medical Intensive Care Unit, Petie Salpetriere Hospital, Sorbonne University, Paris, France; g Assistant Director of Nursing, Sheikh Khalifa Medical City, Abu Dhabi, UAE; h Senior Research Fellow, Acute & Critical Care, School of Public Health and Preventive Medicine, Monash University, Melbourne, Australia; i Executive Director, Monash Partners Academic Health Science Centre, Head of the Division of Clinical Trials and Cohort Studies, School of Public Health and Preventive Medicine, Monash University, Deputy Director of the Australian and New Zealand Intensive Care-Research Centre, Monash University, Melbourne, Australia; j Cardiac Surgery, HVTI, Cleveland Clinic, Abu Dhabi, UAE

**Keywords:** life after ECMO, HRQoL, rehabilitation, disability

## Abstract

Extracorporeal membrane oxygenation (ECMO) has become an increasingly vital intervention for patients with severe cardiac and respiratory failure, significantly improving short-term survival rates. However, there is growing recognition of the importance of long-term outcomes, including health-related quality of life (HRQoL), functional recovery, and psychological well-being. This narrative review synthesizes clinical studies, systematic reviews, and cohort analyses from the past 20 years using PubMed and Google Scholar, focusing on adult ECMO survivors.

Studies consistently demonstrate that although many survivors achieve functional independence, a significant proportion experience persistent limitation. Common long-term sequelae include reduced physical capacity, depression, anxiety, cognitive impairment, and challenges in social reintegration such as returning into employment. Outcomes vary by ECMO modality: Veno venous (VV) ECMO survivors typically report better HRQoL than those receiving Veno arterial (VA) ECMO, although depression appears more prevalent among VV ECMO survivors. Significant complications including neurological injury, limb ischemia, and bleeding remain major contributors to morbidity, particularly in VA ECMO cohorts. The differences reflect variation in underlying disease and complication burden between the 2 modalities. Rehabilitation strategies emphasizing early physiotherapy, structured discharge planning, and comprehensive long-term follow-up are associated with improved functional and psychosocial outcomes. Despite rising survival, existing literature reveals considerable heterogeneity, a lack of standardized outcome measures, and limited prospective multicenter data. Future research should focus on innovations in ECMO circuit design, safer anticoagulation protocols, and the development of standardized rehabilitation pathways. Incorporating long-term functional, psychological, and social outcomes into ECMO care paradigms, is essential to move beyond survival towards meaningful recovery, ultimately optimizing quality of life for ECMO survivors.

Extracorporeal membrane oxygenation (ECMO) is a life-support intervention that provides a lifeline for select patients facing severe respiratory or cardiac failure. By temporarily assuming the functions of the heart and lungs via an external circuit, ECMO provides vital organ support, allowing time for recovery and serving as a bridge to definitive treatment or transplantation. It is particularly useful in situations where conventional therapies fail, such as during severe acute respiratory distress syndrome (ARDS) or refractory cardiac failure. Following ECMO decannulation, patients often continue to require intensive care, with recovery extending over weeks to months. If they improve, it is common to have lingering symptoms that may persist for many months after such a severe illness and ECMO treatment, with effects often lasting beyond discharge. Despite survival, many patients experience persistent symptoms that may endure well beyond hospital discharge. These long-term challenges frequently affect physical, psychological, and cognitive domains, potentially complicating recovery. However, with comprehensive support and rehabilitation, many of these impairments gradually improve. This narrative review aims to provide a thorough view of life after ECMO. Looking at variations between the 2 modalities VA ECMO vs VV ECMO as illustrated in Figure AB, and their impact on post-discharge HRQoL, functional status, and psychological well-being. We also discuss current supportive care strategies, and future directions to optimize recovery in this vulnerable population.

**Figure AB:**
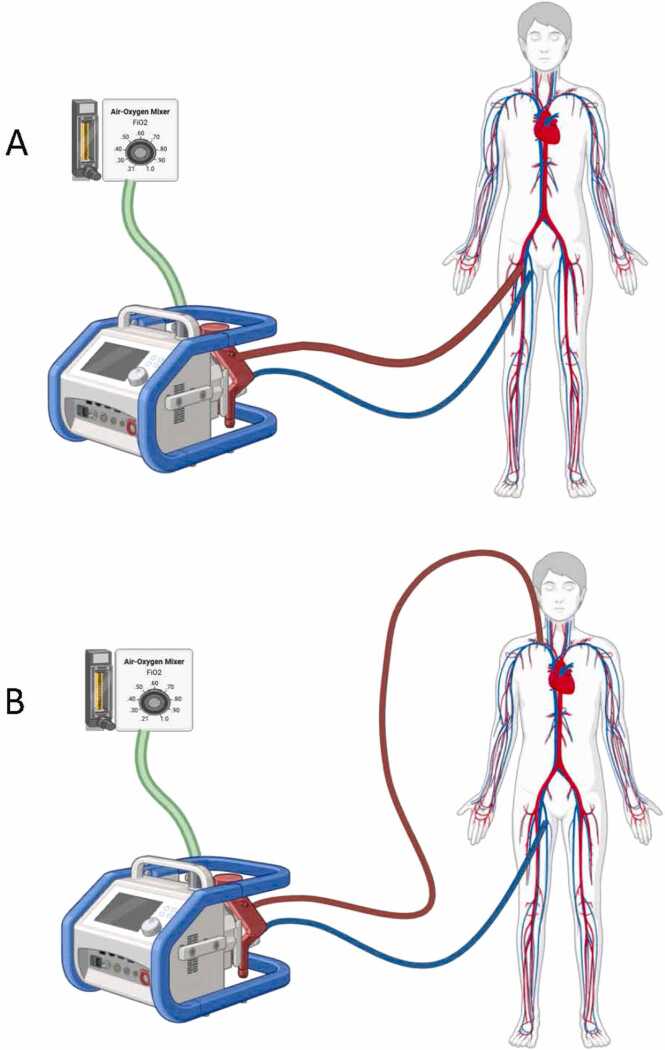
ECMO (A) Veno-arterial (VA) ECMO in a femoral–femoral configuration, with venous drainage from the femoral vein and arterial return to the femoral artery. (B) Veno-venous (VV) ECMO in a femoral–jugular configuration, with venous drainage from the femoral vein and return via the internal jugular vein.

## Method

**Figure 1:**
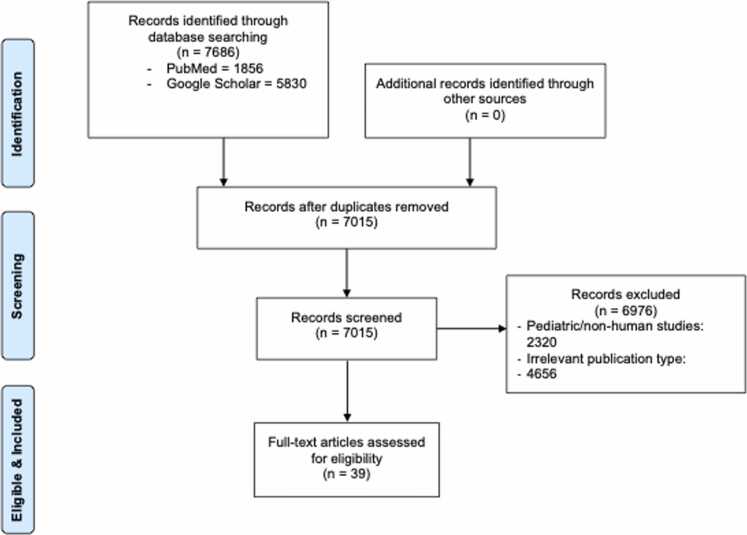
Databases searched PubMed (n = 1856), Google Scholar (n = 5830), 7686 records. After removing duplicates (n = 671), 7015 records were screened. Based on title and abstract, 6976 were excluded for being irrelevant, pediatric, or non-human. Of the full texts reviewed, 39 met eligibility criteria. Exclusions included non-English studies, animal studies, and those lacking outcome data or procedural success.

A comprehensive literature search was conducted to identify clinical studies, trials, and case reports addressing long-term outcomes in adult ECMO patients, with a focus on HRQoL, functional status, and psychological well-being over the past 20 years. Electronic databases, including PubMed and Google Scholar were systematically searched to capture high-impact studies relevant to post-ECMO HRQoL, disability, and psychological sequelae. The search employed a combination of Medical Subject Headings (MeSH) and keywords such as “extracorporeal membrane oxygenation,” “ECMO,” “quality of life,” “HRQoL,” “functional outcomes,” “disability,” “long-term outcomes,” and “mental health.” The strategy prioritized systematic reviews, meta-analyses, and large cohort studies published within the last 15 years to ensure inclusion of recent, high-quality evidence. Inclusion criteria encompassed adult patients (≥18 years) who underwent VV, VA, or extracorporeal cardiopulmonary resuscitation (eCPR) and survived to hospital discharge. Studies involving ventricular assist devices without oxygenation, preclinical or animal models, and pediatric populations were excluded. Additionally, filters were applied to restrict results to human studies published in English. Following identification, relevant articles underwent data extraction capturing study design, patient demographics, ECMO modality, and support characteristics, and clinical outcomes. An integrative synthesis approach was employed to consolidate findings from diverse sources, facilitating a comprehensive analysis of long-term functional, psychological, and quality of life outcomes following ECMO (Figure 1).

## Results

### Clinical outcomes

According to the Extracorporeal Life Support Organization (ELSO) registry, based on the data from 570 centers, more than 219,301 patients received ECMO through the end of 2023 and were recorded in the registry. The number of ECMO cases increased substantially in the last 10 years, from 3446 in 2010 to nearly 19,000 in 2023. Moreover, the number of ECMO centers reporting to ELSO tripled in that period.^1^ The reported survival rate of all adult ECMO patients was 67%, with a 54% survival to hospital discharge or transfer.^2^

Hodgson et al assessed incidence of death or disability associated with ECMO up to 6 months after treatment. A prospective multicentre study was done at 23 hospitals in Australia between 2019 and 2020. The study included all adults from the Extracorporeal Life Support (ECLS) registry embedded cohort (EXCEL study) receiving ECMO support for either respiratory failure, cardiac failure, or cardiac arrest. Patients who received a WHO Disability Assessment Schedule 2.0 (WHODAS) score of > 25% met the primary outcome of incidence of death or disability. Health status EuroQol 5-dimension, 5-level questionnaire (EQ-5D-5L) and EuroQol Visual Analogue Scale (EQ-VAS) tests were used to measure health status, Instrumental Activities of Daily Living (IADL) test, and the Barthel index was used to measure independence, and the Montreal Cognitive Assessment (MoCA-BLIND) test was used to measure cognitive dysfunction.

From 391 patients, the primary outcome of death or moderate-to-severe disability was found in 260 (66%) patients. Of those, 202 patients received VA ECMO and 136 (67%) met the primary outcomes of death or disability. One hundred eleven patients received VV ECMO, and 64 patients received eCPR, of which 60 patients (54%) and 64 patients (83%) met the primary outcome for VV ECMO and eCPR, respectively. After adjustment, death or moderate-to-severe disability was higher in patients who received eCPR over those who received VV-ECMO, but not found in eCPR over VA ECMO.

In the study, one third of patients were without death, or moderate to severe disability. The remaining did not survive or had physical, psychological, and cognitive functioning affected. This study highlights further studies are needed to understand the 180-day and longer-term prognosis of patients with different diagnoses receiving different modes of ECMO.^3^

### Quality of life

Survivors of severe cardiac and respiratory disease, including ARDS, cardiogenic shock (CS), and post-operative heart failure, often experience persisting physical, psychological, social, and functional impairments following hospital discharge. With advances in ECMO and improved survival rates, outcome evaluation should extend beyond short-term mortality to encompass long-term recovery and well-being. HRQoL, defined as perceived physical and mental health over time, is frequently measured using validated questionnaires in ECMO survivors.^3^

Hodgson et al identified discrepancies between disability and HRQoL, shaped by resilience, adaptation, and environmental support. Importantly, HRQoL scores may not capture functional limitations such as inability to return to work, thereby risking underestimation of patient and family burden.^3^ A systematic review of VV ECMO for ARDS reported that 265 survivors showed lower HRQoL (SF-36, EQ-5D), as compared to healthy controls. However, outcomes were similar or superior to those of patients receiving conventional ARDS treatment. Notably, more than half of VV ECMO survivors returned to work, compared with fewer patients in conventional treatment cohorts. However, smaller cohort data suggest less favorable functional recovery. In a retrospective study, Hodgson et al reported reduced HRQoL among 15 survivors of ARDS treated with ECMO, with only 26% returning to pre-illness employment and over half experiencing persistent mobility problems. It remains unclear whether these deficits can be attributed to ECMO itself or to the severity of ARDS.^4^ Nevertheless, physical impairments, often linked to prolonged cannulation, were frequent,^5^, ^6^ underscoring the importance of post-ECMO physiotherapy and rehabilitation.

Psychological outcomes among ECMO survivors remain a significant concern. A cohort study demonstrated higher depression rates in VV compared with VA ECMO survivors, with ECMO modality identified as an independent predictor of post-discharge depression.^7^ This may reflect prolonged mechanical ventilation, sedation, and immobility in VV ECMO, whereas VA ECMO survivors, despite greater illness severity, often experience lower employment rates, likely related to older age and functional limitations associated with femoral cannulation. Pre-existing socioeconomic disadvantage and prior psychiatric morbidity further influence outcomes. Another study also supports this distinction, demonstrating a higher prevalence of neuropsychiatric symptoms in VV compared with VA ECMO survivors (55% vs 32%), although no differences were observed in neurocognitive or functional outcomes between modalities.^8^ However, evidence directly comparing VV and VA ECMO remains limited. Most studies report psychological outcomes across mixed ECMO populations, consistently demonstrating a substantial burden of depression, anxiety, and post-traumatic stress symptoms among survivors. These findings underscore the need for structured psychological assessment and follow-up in all ECMO survivors.

Meta-analyses and long-term studies further highlight differences between VV and VA ECMO populations. A systematic review of 913 patients found VV patients achieved slightly better long-term HRQoL, given early recognition and management of both physical and psychological sequelae.^9^, ^10^ Wilcox et al. found ARDS ECMO survivors had lower HRQoL but reduced psychological morbidity relative to those managed with conventional ventilation.^11^ Bursa et al (37 studies) reported that respiratory failure patients on ECMO, mostly returned home or to work, although anxiety, depression, and mild pulmonary deficits were common.^12^ In contrast, VA ECMO survivors show more variable and often poorer long-term outcomes. A Norwegian study of 20 predominantly VA ECMO survivors reported 75% achieved normal HRQoL at 6.5 years. However, survivors of CS and ECPR had the lowest scores.^13^, ^14^ Conversely, long-term VV ECMO cohorts in a study of 289 patients reported favorable HRQoL with minimal limitations.^15^ Taken together, these findings suggest that although HRQoL may recover in a proportion of ECMO survivors, particularly in VV populations, it may underestimate persistent functional impairment, especially in VA ECMO cohorts. While survival has improved, understanding the quality of that survival remains essential.

Key findings from HRQoL studies and long-term outcomes after ECMO are summarized in Table 1, demonstrating reduced physical functioning compared with healthy controls and more variable mental health recovery. VV ECMO survivors generally achieve better HRQoL than VA ECMO survivors, although return-to-work rates and functional independence remain limited across both groups.

**Table 1:** Studies on HRQOL & Long-term Outcomes After ECMO

**Author**	**Type of study**	**Study population**	**Results/findings**	**Conclusion**
Hodgson et al^3^	Observational	391 Patients	HRQOL alone did not fully reflect patient outcome. New/severe disability was not represented. Influenced by resilience, adaptation, and environment. While reduced productivity and inability to return to work was noted, other disability burdens were not captured	HRQoL alone may not fully capture important functional limitations; broader, multidimensional assessment tools are required for evaluation of long-term outcomes.
Kurniawati ER et al^5^	Systematic review	265 VV ECMO survivors (ARDS); 335 compared with conventional treatment	VV ECMO survivors demonstrated reduced HRQoL compared with general population, particularly in physical domains. Outcomes were broadly comparable to conventionally treated ARDS patients, although heterogeneous findings across studies. Persistent functional limitations observed despite acceptable HRQoL scores in some cohorts.	HRQoL following VV ECMO is impaired compared with the general population but broadly comparable to conventionally treated ARDS patients; persistent functional limitations remain despite acceptable HRQoL scores.
Zeng X et al^6^	Single centered cross-sectional study	Adult ECMO survivors (mixed VV and VA ECMO), long-term follow-up	Lower HRQoL scores in physical functioning, bodily pain, vitality, and role-emotional vs general population.	Long-term HRQoL following ECMO is impaired and heterogeneous, with persistent multidimensional deficits. Recovery is influenced by multiple factors, and HRQoL alone may not fully reflect overall functional status.
Lin WJ^7^	Observational	142 ECMO survivors (VV vs VA)	Depression scores were significantly higher in VV ECMO survivors compared with VA ECMO (*p* = 0.018). ECMO modality was an independent predictor of post-discharge depression (*p* = 0.008), with VV ECMO associated with greater psychological burden.	VV ECMO survivors may experience a higher burden of post-discharge depression, highlighting the need for structured psychological assessment and multidisciplinary follow-up.
Knudson KA^9^		Adult ECMO survivors (VV and VA ECMO) across 31 studies with heterogeneous populations and follow-up durations	Long-term HRQoL was generally reduced compared with population norms, with the greatest impairment in physical domains. Findings across studies were heterogeneous, and while some variation between VV and VA ECMO was reported, this was not consistent.	Early identification and management of physical and mental health problems may improve long-term recovery in ECMO survivors.
Hodgson et al^4^	Single-centre, observational follow-up study	Adult ARDS patients treated with VV ECMO −15 survivors at a median of ∼8 months	Survivors demonstrated reduced HRQoL compared with healthy controls, particularly in physical function, vitality, social function, and mental health. Psychological morbidity was also frequent, with moderate to severe anxiety and depression reported in a substantial proportion. Functional recovery was incomplete, with only ∼50% returning to work and only 26% returning to previous work levels.	Despite good survival, ECMO survivors experience reduced HRQoL and persistent physical and psychological impairment. These findings highlight the importance of long-term follow-up and support, and demonstrate that short-term outcomes do not reflect long-term recovery.
Wilcox et al^11^	Systematic review (6 studies; 1 RCT, 5 observational studies)	Critically ill adult patients with ARDS/acute respiratory failure across 6 studies (n = 245 for HRQoL comparison; n = 217 for psychological outcomes)	ECMO survivors demonstrated greater reductions in HRQoL compared with conventionally mechanically ventilated patients (SF-36 difference 5.40). However, ECMO survivors had significantly lower psychological morbidity, with reduced depression and anxiety compared to conventional treatment.	Evidence on long-term outcomes following ECMO remains limited, and further studies are required to confirm findings and identify prognostic factors associated with improved recovery.
Bursa et al^12^	Review (37 studies)	Respiratory failure patients post-ECMO	Many survivors returned to independent living and work, but persistent psychological morbidity, including anxiety, depression, and sleep disturbances, remained common. Pulmonary limitations were generally mild and tended to improve over time.	Most ECMO survivors return to independent living and work; however, long-term morbidity remains significant, with persistent psychological symptoms and mild pulmonary limitations that may improve over time.
Norwegian study^13^	Cross-sectional, long-term	Adult ECMO patients treated at a single centre (n = 74; predominantly VA ECMO 87%), with long-term survivors surveyed (n = 20) at a mean follow-up of 6.5 years	Most survivors lived independently and were self-sufficient, with approximately 50% returning to work. HRQoL scores were generally within the lower normal range compared with population norms. However, impairments persisted in specific domains, including general health, physical, and emotional role functioning. A substantial proportion reported ongoing problems with daily activities, mobility, pain, and psychological symptoms. HRQoL improved with increasing time since ECMO.	Long-term HRQoL in ECMO survivors is generally acceptable, although a subset experience persistent functional and psychological limitations. HRQoL improves over time, and identification of patients at risk for poorer recovery may help guide rehabilitation strategies.
Tramm et al^14^	Single-centre retrospective cohort study	Adult ECMO patients treated at a single centre (n = 74; predominantly VA ECMO 87%), with long-term survivors surveyed (n = 20) at a mean follow-up of 6.5 years.	Among long-term survivors, most lived independently and were self-sufficient, with approximately 50% returning to work. However, a substantial proportion reported persistent impairments, including problems with daily activities (40%), mobility (35%), anxiety/depression (35%), and pain/discomfort (55%). Lower HRQoL was associated with psychological distress, cognitive complaints, and functional limitations. HRQoL improved with increasing time.	Adult ECMO patients treated at a single centre (n = 74; predominantly VA ECMO 87%), with long-term survivors surveyed (n = 20) at a mean follow-up of 6.5 years.
Rilinger et al^15^	Retrospective study	289 VV ECMO patients; 53 completed surveys	Long-term survival was high, with most survivors returning to work (82%). HRQoL was comparable to the general population, with only mild physical limitations and favorable psychological outcomes with minimal pulmonary/mental limitations.	VV ECMO survivors demonstrate encouraging long-term survival and high health-related quality of life, with only minor physical and pulmonary limitations, indicating a favorable long-term prognosis after severe ARDS.

**Figure 2:**
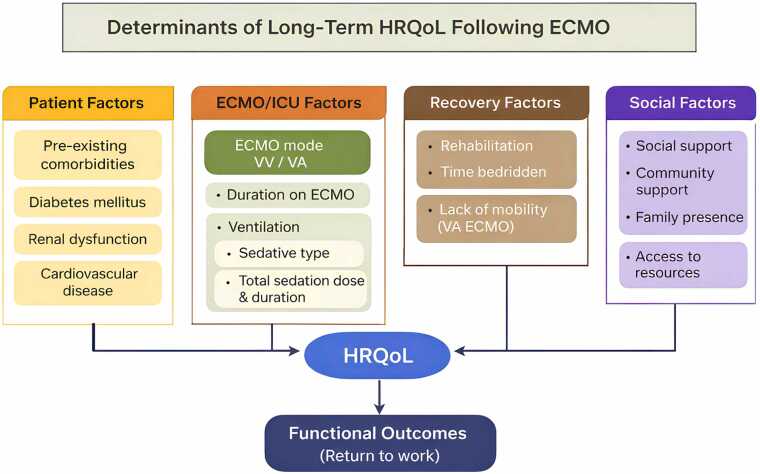
Determinants of long-term HRQoL following ECMO.

Figure 2 contextualizes these findings, showing that outcomes arise from the interaction between patient factors, ECMO-related variables, rehabilitation, and social support, rather than a single determinant.

### Modality-specific outcomes VV vs VA ECMO

Long-term outcomes following ECMO differ according to modality, reflecting differences in underlying disease, patient characteristics, and complication profiles. Notably, the literature on VV ECMO is more robust for long-term HRQoL outcomes, whereas VA ECMO literature more commonly focuses on complications and functional disability. VV ECMO is predominantly used in patients with severe but potentially reversible respiratory failure, whereas VA ECMO is typically employed in patients with cardiogenic shock or cardiac arrest, frequently in the context of underlying cardiovascular disease. These differences in indication strongly influence both complication patterns and long-term recovery trajectories.

VV ECMO survivors generally demonstrate more favorable long-term HRQoL compared with VA ECMO cohorts, although this finding is modest and heterogeneous across studies.^5^, ^9^, ^11^ Despite this, persistent physical impairment, reduced exercise capacity, and delayed return to work remain common, reflecting the broader impact of critical illness and post-intensive care syndrome.^5^, ^16^ Importantly, long-term data from ARDS survivors demonstrate that functional limitations may persist despite recovery of pulmonary function, suggesting that part of the long-term burden observed in VV ECMO patients relates to severe respiratory failure rather than ECMO exposure alone.

Complications in VV ECMO are largely related to anticoagulation and circuit dynamics, including bleeding, circuit thrombosis, and oxygenator failure.^17^ Neurological complications also occur, with intracranial hemorrhage representing a key concern in VV ECMO. Some studies suggest that anticoagulation exposure and rapid correction of hypercapnia during ECMO initiation may contribute to cerebral haemodynamic instability and increased risk of intracranial bleeding, representing a key area where clinicians can adjust.^18^ In addition, prolonged mechanical ventilation and critical illness contribute to physical deconditioning and long-term functional impairment.

In contrast, VA ECMO survivors experience a greater burden of complications and long-term morbidity. Vascular complications are a defining feature of VA ECMO, driven by arterial cannulation, and include limb ischemia, arterial injury, and distal hypoperfusion, which may result in long-term disability or amputation.^19^, ^20^ Bleeding is also more frequent in VA ECMO and is associated with increased mortality and multiorgan complications.^19^ Neurological injury, including ischemic stroke and hypoxic brain injury, is more prevalent in VA ECMO populations, particularly among patients requiring eCPR.^18^, ^21^ These factors contribute to higher rates of functional limitation and disability, even among survivors.

The mechanisms underlying these modality-specific differences are multifactorial. In VA ECMO, systemic perfusion reflects the interaction between pump flow and native cardiac output, and the presence of dual circulation haemodynamics may contribute to cerebral and systemic perfusion abnormalities and increase the risk of neurological injury.^22^ In contrast, VV ECMO primarily supports gas exchange while preserving native haemodynamics, reducing the risk of systemic hypoperfusion but exposing patients to complications related to prolonged respiratory failure, anticoagulation, and critical illness. These physiological differences likely contribute to the observed variation in long-term outcomes between modalities.

Importantly, the relationship between ECMO modality and long-term outcome is not uniform across studies. While VV ECMO is often associated with better HRQoL, several cohorts report persistent physical and psychological impairment despite acceptable global HRQoL scores.^9^, ^11^ Conversely, VA ECMO survivors may report reasonable quality of life despite significant functional limitation, highlighting a discrepancy between patient reported outcomes and objective measures of recovery. These inconsistencies likely reflect differences in patient selection, follow-up duration, outcome measures, and underlying disease severity.

From a clinical perspective, these findings support a modality-specific approach to management and follow-up. In VA ECMO, early recognition and prevention of vascular complications are critical, including routine assessment of limb perfusion and consideration of distal perfusion strategies. Enhanced neurological monitoring and early neurocognitive assessment are also essential given the high burden of neurological injury. In VV ECMO, careful management of anticoagulation and avoidance of rapid PaCO₂ correction may reduce neurological risk, while early mobilization and structured rehabilitation are key to addressing long-term physical deconditioning.

Across both modalities, survival alone should not be considered an adequate outcome measure. Structured follow-up should incorporate assessment of functional status, cognitive recovery, and return to work, with integration of multidisciplinary rehabilitation and psychological support. Overall, a modality-specific, patient-centered approach to ECMO care is essential to optimize long-term recovery and quality of life.

**Figure 3:**
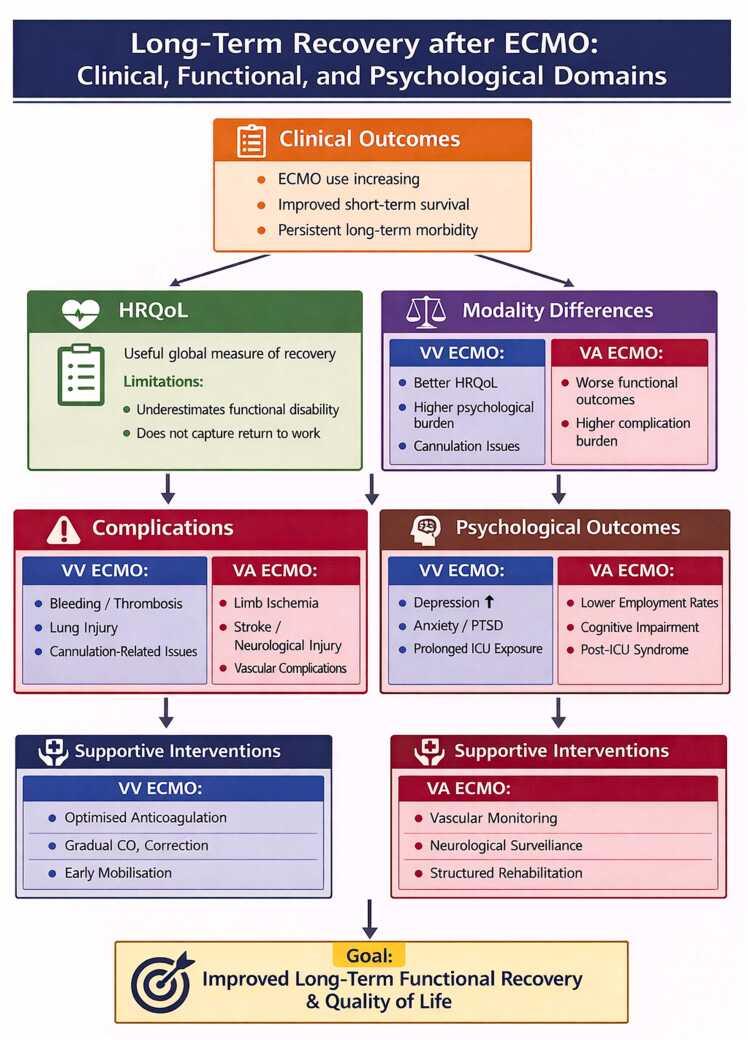
Long-term recovery after ECMO: clinical, functional, and psychological domains.

An overview of the key determinants of long-term outcomes following ECMO, including modality-specific differences, complications, and recovery pathways, is presented in Figure 3.

### Complications and challenges

The differences in long-term outcomes between VV and VA ECMO could largely be driven by modality-specific complication profiles. ECMO practice remains heterogeneous, with significant variability in patient selection, management, and outcome reporting, particularly in VA ECMO. This heterogeneity, combined with reliance on short-term mortality outcomes, limits comparison across studies and obscures long-term recovery trajectories.

Complication profiles differ between ECMO modalities and are key determinants of long-term outcomes. Bleeding remains the most frequent complication (27.6%), occurring more commonly in VA ECMO due to arterial cannulation and pre-existing surgical exposure, and is strongly associated with increased morbidity, multiorgan dysfunction, and mortality.^23^ Vascular complications, affecting approximately 30% of patients, are also predominantly observed in VA ECMO and include limb ischemia, thromboembolism, and arterial injury, with risk influenced by patient comorbidities and cannulation strategy.^19^, ^20^

Neurological complications occur in 10% to 20% of ECMO patients and represent a major contributor to long-term disability. These differ by modality, with ischemic stroke and hypoxic brain injury more common in VA ECMO, particularly in cardiogenic shock and eCPR populations, while intracranial hemorrhage is more frequently observed in VV ECMO. Mechanistically, factors such as coagulopathy, thrombocytopenia, and rapid PaCO₂ shifts may contribute to cerebral injury, highlighting potential modifiable risks.^18^

Importantly, these complications are not isolated events but directly influence long-term functional recovery. Despite comparable HRQoL to conventional care in some cohorts, many ECMO survivors experience persistent disability, reduced exercise capacity, and incomplete return to work, reflecting the cumulative burden of complications and critical illness.^16^, ^17^, ^24^

The spectrum of ECMO-related complications and their impact on outcomes are summarized in Table 2. Bleeding and vascular complications remain the predominant adverse events, particularly in VA ECMO, and are closely associated with poorer long-term functional outcomes. The spectrum of ECMO-related complications and their association with long-term outcomes are summarized in Table 2, highlighting key modality-specific risks.

**Table 2:** Complications and Challenges With ECMO

**Author**	**Type of study**	**Study population**	**Results/findings**	**Conclusion**
Burrell et al^25^	Systematic review	46 VA ECMO studies including 20,375 patients (predominantly retrospective, observational studies)	Forty-six retrospective studies (20,375 patients) showed marked heterogeneity, with inconsistent reporting of selection criteria, ECMO management, outcomes, and complication definitions; mortality was the most common outcome and bleeding the most frequently reported complication.	VA ECMO evidence is limited by inconsistent reporting and non-standardized definitions, restricting comparability; consensus-based definitions and patient-centered outcomes are needed.
Willers et al. (ELSO registry)^23^	Registry analysis	(20 years) Adult ECLS patients (n = 53,644; VV = 19,748, VA = 30,696)	Bleeding occurred in 27.6%, more frequent in VA, associated with higher mortality; rates decreased over time, mainly from reduced cannulation/surgical bleeding.	Bleeding has decreased but remains common and linked to increased mortality; causes are multifactorial and require further study
Jia, Deng et al^19^	Systematic review and Meta-analysis	VA vs VV ECMO 47 studies (n = 6583 ECMO patients; predominantly VA ECMO)	Vascular complications occurred in ∼29.5%, more frequent in VA ECMO; bleeding and limb ischemia were most common, with ∼46% survival to discharge.	Vascular complications are common (∼30%), especially in VA ECMO; distal perfusion cannulas reduce limb ischemia and mortality.
Nationwide study^20^	Nationwide registry	6968 ECMO patients	114 limb amputations, mainly VA ECMO. Risk higher in cardiovascular disease and CHF. Norepinephrine use lowered risk.	Limb amputation is rare among ECMO survivors and not linked to increased long-term mortality; further study on quality-of-life impact is needed.
Kalra et al^21^	Systematic review	(59 studies) 3298 ECMO patients (86% VA, 14% VV)	Long-term neuropsychiatric, cognitive, and functional impairments frequent, especially in VA/ECPR patients. Assessment methods varied.	Neurological sequelae are common; standardized assessment methods needed.
Bogotá case series^26^	Retrospective single centre	Post-cardiac arrest patients with ROSC treated with ECMO (n = 32)	Survival 71.8% with favorable neurological outcomes (CPC 1, mRS 2, Barthel 75) and acceptable HRQoL at 6 months; no VA vs VV difference	ECMO after cardiac arrest with ROSC shows good survival and neurological outcomes; not a contraindication with appropriate selection.
Zhang et al^18^	Narrative review	Adult ECMO patients (VA and VV) across multiple studies	Neurologic complications are common and associated with increased mortality; VA ECMO has higher rates, with AIS predominant in VA and ICH in VV. Long-term survivors may develop cognitive and psychological impairment.	Neurologic complications significantly impact outcomes; early recognition, multimodal monitoring, and prevention strategies are essential to improve survival and quality of life.
Turgeon J et al^16^	Systematic review	ARDS patients treated with ECMO(1 RCT, 31 observational)	No difference in HRQoL, cognitive, mental, or functional outcomes vs CMV; high disability, with 49% returning to work and 23% requiring assistance.	ECMO survivors have significant long-term disability across multiple domains; further comparative studies are needed.
Teijeiro-Paradis R et al^24^	Narrative Review	Patients with severe respiratory failure receiving VV ECMO	VV ECMO is associated with multiple complications across care, including bleeding, thrombosis, neurologic and renal injury, infections, and circuit issues; long-term sequelae include reduced HRQoL and functional impairment. Term sequelae common.	ECMO-related complications are influenced by patient selection, timing, and management; careful decision-making is essential to minimize harm and improve outcomes.
Vaquer S,^17^	Systematic review	Severe ARDS on VV ECMO. 1042 patients)	Hospital mortality ∼38%; complications ∼40% (bleeding most common ∼29%); complication-related mortality low (∼7%).	VV ECMO shows reduced mortality despite severe illness; complications are common but have limited impact on mortality.

### Rehabilitation

Physiotherapy is integral to recovery in ECMO patients, supporting early mobilization, pulmonary hygiene, and long-term functional improvement.^27^ During the COVID-19 pandemic, structured physiotherapy programs proved especially valuable, improving cooperation, muscle strength, and daily activity performance, with benefits continuing after decannulation.^28^ Early mobilization also mitigates post-intensive care syndrome (PICS), characterized by persistent physical, cognitive, and psychological impairments. Studies in both ECMO and broader ICU populations have shown improved physical functioning and exercise tolerance, as reflected by higher scores on the SF-36 Physical Functioning Scale and 6-min walk tests. Fernando et al. further demonstrated that ECMO survivors had a modestly higher incidence of new mental health or social diagnoses compared with non-ECMO ICU patients, underscoring the importance of integrating psychosocial support into rehabilitation.^29^

From a clinical perspective, several practical strategies can be implemented. Early mobilization protocols should be initiated as soon as hemodynamically feasible, including passive range-of-motion exercises, in-bed cycling, and progression to active mobilization. Multidisciplinary rehabilitation involving physiotherapists, occupational therapists, and psychological support services should be incorporated into routine care. Functional assessment should extend beyond discharge, with evaluation of mobility, independence in daily activities, and return to work.

Rehabilitation needs may differ between ECMO modality, with VA ECMO patients requiring greater emphasis on recovery from vascular and neurological complication, including limb function assessment and neurocognitive screening. While VV patients may benefit from pulmonary and function reconditioning.^5^, ^11^, ^18^, ^19^, ^20^, ^21^, ^27^, ^28^

The importance of structured rehabilitation and long-term follow-up is supported by the literature summarized in Table 3, which highlights the high prevalence of post-intensive care syndrome, including cognitive, psychological, and functional impairment, and emphasizes the need for multidisciplinary, patient-centered recovery pathways

**Table 3:** Summarizes Key Studies Evaluating Post-ECMO Rehabilitation and Long-term Outcomes

**Author**	**Type of study**	**Study population**	**Results/findings**	**Conclusion**
Shirejini et al^30^	Narrative review	ECMO patients (anticoagulation focus)	Bleeding (20%-40%) and thrombosis (10%-46%) are common; driven by circuit-related factors and anticoagulation, with multiple monitoring methods required.	Optimal anticoagulation requires multimodal monitoring; novel agents and real-time techniques are needed to balance bleeding and thrombosis.
Higa et al^31^	Systematic review	27 studies, 3271 VA ECMO patients	High burden of PICS: persistent physical, mental (anxiety 35%, depression 26%, PTSD 21%), and functional impairment; ≤50% return to work; outcomes heterogeneous.	VA ECMO survivors have significant long-term multidomain impairment; outcomes are understudied, requiring prospective and standardized research
Hasasna et al^32^	Single-centre retrospective cohort study	53 VV ECMO survivors followed up to 3 years	QoL acceptable: mental scores similar to general population; physical scores initially lower but normalize by 3 years; higher resource use linked to poorer QoL.	Survivorship programs with rehabilitation and counseling improve QoL and may enhance long-term recovery and cost-effectiveness
Khan et al^33^	Editorial /commentary	59 studies, 3280 ECMO patients (predominantly VA ECMO)	Advocated for comprehensive survivorship programs including multidisciplinary support, rehabilitation, and psychosocial resources.	Focus on holistic, patient-centered recovery.
Research perspective^34^	Commentary/proposal	ECMO patients and rehabilitation networks	Highlights evolving role of ECMO beyond a bridge therapy, including long-term outcomes, patient selection, and system-level considerations.	ECMO should be viewed as part of a broader care continuum, with emphasis on patient selection, long-term outcomes, and integrated care pathways.

These data support the role of early mobilization and structured follow-up in improving functional recovery and HRQoL

### Clinical implications

The findings of this review support a modality-specific approach to ECMO care. In VA ECMO, priority should be given to early detection of vascular complications and structured neurological monitoring. In VV ECMO, attention should focus on minimizing modifiable risks, including avoidance of rapid hypercapnia correction and optimization of anticoagulation strategies. Across both modalities, early mobilization and structured rehabilitation are essential, alongside follow-up assessing functional recovery, cognition, and return to work. These findings emphasize the need to shift from survival-focused care to recovery-focused care in ECMO populations.

### Future directions

Future efforts should focus on addressing key limitations in ECMO care and long-term recovery. Optimization of anticoagulation remains a priority, as thrombosis and bleeding continue to represent major challenges. Achieving effective anticoagulation requires a multimodal approach, including improved monitoring strategies, development of novel anticoagulants, and advances in circuit biocompatibility and surface engineering.^30^

Beyond the acute phase, ECMO survivors frequently experience post-intensive care syndrome, including neurocognitive and psychological sequelae such as memory impairment, depression, and post-traumatic stress disorder. These effects may be particularly pronounced in VA ECMO populations due to higher rates of neurological injury. Standardized screening tools and multicentre prospective studies are needed to better characterize long-term outcomes and guide targeted rehabilitation strategies.^31^, ^32^

Comprehensive programs incorporating multidisciplinary care, including physical rehabilitation, psychological support, and social reintegration, are essential to improve long-term recovery.^33^ Future research should prioritize the development of structured rehabilitation pathways and standardized follow-up protocols, alongside evaluation of individualized exercise and recovery programs.^34^

Overall, advancing ECMO care will require a shift towards integrated, patient-centered models that address both acute management and long-term recovery, ensuring that improvements in survival translate into meaningful functional and psychological outcomes.

## Summary

ECMO has transformed the management of severe cardiac and respiratory failure, improving short-term survival. However, long-term outcomes among survivors remain suboptimal. HRQoL is frequently diminished, with a growing recognition that standardized measures may underestimate the true burden of survivorship. Discrepancies between HRQoL scores and lived experience often reflect unmeasured factors such as loss of productivity, caregiver burden, and ongoing psychosocial distress. Major complications, including bleeding, vascular injury, limb ischemia, and neurological sequelae such as stroke, cognitive dysfunction, depression, anxiety, and post-traumatic stress disorder (PTSD), remain key determinants of long-term morbidity. These outcomes differ between ECMO modalities, with VA ECMO associated with a greater burden of complications and functional disability, while VV ECMO survivors, although demonstrating comparatively better HRQoL, continue to experience significant physical and psychological impairment. Supportive strategies, including early physiotherapy, structured rehabilitation, and standardized follow-up, improve recovery but remain inconsistently applied across centers. Psychological morbidity is increasingly recognized as a major component of post-ECMO recovery, reinforcing the need for integrated multidisciplinary care. Despite improving survival, substantial knowledge and practice gaps persist. Current evidence is limited by retrospective designs, heterogeneity in patient populations, inadequate baseline assessments, and a lack of standardized outcome measures.

Future priorities include multicentre prospective studies, optimization of anticoagulation strategies, improved monitoring of complications, and development of structured rehabilitation and follow-up pathways. Ultimately, a multidisciplinary, patient-centered approach addressing the physical, psychological, and social dimensions of recovery is essential to ensure that survival following ECMO translates into meaningful, sustained quality of life.

## Limitations

This review highlights several important considerations that also inform future research directions. The current evidence base reflects diverse patient populations, ECMO indications, and management strategies, which, while enriching the field, can limit direct comparability between studies. Much of the available data is derived from retrospective and single-centre studies, underscoring the opportunity for more robust prospective, multicentre research. Outcome assessment remains variable, particularly for HRQoL, functional status, and neurocognitive recovery, and pre-ECMO baseline data are often lacking, making attribution of long-term effects more complex. Follow-up duration is also inconsistent, with limited data extending beyond the first year post-ECMO. Additionally, key domains such as neurological, bleeding, vascular, and ethical considerations are not uniformly integrated into long-term outcome analyses. Finally, as a narrative review, this study reflects the available published literature and may not capture all emerging or unpublished data. These factors collectively emphasize the need for standardized outcome measures and comprehensive longitudinal studies to further advance the understanding of recovery after ECMO.

## Conclusion

ECMO has transformed the management of severe cardiac and respiratory failure, improving short-term survival. However, long-term recovery remains incomplete, with many survivors experiencing persistent physical, cognitive, and psychological impairment. These outcomes differ between modalities, with VA ECMO associated with a greater burden of complications and disability, while VV ECMO survivors, although demonstrating comparatively better HRQoL, still experience significant functional limitations.

Importantly, these differences appear to be driven less by ECMO modality itself and more by the underlying disease, complication burden, and recovery trajectory. This highlights the need to shift focus from survival alone to meaningful recovery. A structured, multidisciplinary approach incorporating early rehabilitation, complication prevention, and long-term follow-up is essential to optimize outcomes. Future efforts should focus on improving patient selection, reducing complication burden, and developing standardized pathways for post-ECMO care.

through improved anticoagulation strategies, advanced monitoring technologies, individualized rehabilitation protocols, and structured long-term follow-up. A multidisciplinary, patient-centered approach is critical to ensure that survival is accompanied by meaningful, sustained quality of life for ECMO survivors.

## Conflicts of interest

Dan Brodie previously consulted for LivaNova. He has been on the medical advisory boards for Medtronic, Inspira, Cellenkos, HBOX Therapies and Vantive. He is the President of the Extracorporeal Life Support Organization (ELSO) and the Chair of the Board of the International ECMO Network (ECMONet), and he writes for UpToDate.

## Declaration of Competing Interest

The authors declare that they have no known competing financial interests or personal relationships that could have appeared to influence the work reported in this paper.
